# Potentially Inappropriate Medication Among People With Dementia in China: A Nationwide Cross-Sectional Study

**DOI:** 10.3389/fphar.2022.929584

**Published:** 2022-06-13

**Authors:** Mengnan Zhao, Zhaoyan Chen, Fangyuan Tian, Ting Xu

**Affiliations:** ^1^ Department of Pharmacy, West China Hospital, Sichuan University, Chengdu, China; ^2^ West China School of Medicine, Sichuan University, Chengdu, China

**Keywords:** potentially inappropriate medications, dementia, outpatient, older, psychotropic drugs

## Abstract

**Objectives:** The purpose of this study was to explore the prevalence of potentially inappropriate medication (PIM) among older outpatients (age ≥ 65 years old) with dementia in eight cities in China using the AGS Beers criteria of 2019 and to identify the potential factor increasing the number of PIMs.

**Methods:** A cross-sectional study about PIM in older outpatients with dementia from January 2020 to December 2020 was carried out in eight cities in China, Chengdu, Beijing, Guangzhou, Shanghai, Shenyang, Tianjin, Zhengzhou, and Hangzhou, distributing five major geographical regions in China (east, west, north, south, central). The diagnosis of dementia was based on the International Classification of Diseases (ICD-10) to identify. Based on the 2019 AGS Beers criteria, the PIM prescriptions were evaluated. The identification of potential factors was completed using a binary logistic regression model.

**Results:** Of 18,624 older outpatients with dementia, 3.52% were detected with 1 PIM, and 35.91% received at least two PIMs. The antipsychotic drugs quetiapine and olanzapine were most frequently prescribed in patients with PIM, accounting for 8.01 and 7.36%, respectively. Logistic regression analyses showed that female patients with dementia aged >80 years who took more medications were exposed easily to PIM use.

**Conclusion:** PIM use among older outpatients with dementia in China is highly prevalent, and the associated risk factors were increasing age, female sex, and number of medications. The most frequently prescribed drugs by clinicians were anpsychotropic drugs, which were much more frequent than other drugs.

## Introduction

Dementia is a degenerative disease accompanied by impaired cognitive function. According to statistical data from the World Health Organization (WHO), approximately 50 million people are diagnosed with dementia worldwide, and 60% live in low- and middle-income countries. By 2050, the number is up to 139 million (WHO, 2020). Due to the aging global population, the growing trend will be more apparent, especially in developing countries (Prince et al., 2015; Banta et al., 2017). A study showed that the prevalence of dementia is linked with age, and females are more susceptible to developing dementia than males (Rizzi et al., 2014). As the most populous country and the largest developing country in the world, China has a large number of dementia patients. A study performed by Jia LF showed that the prevalence of dementia in China is estimated to be 6%, and approximately 15.07 million people (age ≥60 years old) have dementia in China (Jia et al., 2020).

Potentially inappropriate medication is a global concern, especially for older people with reduced physical function. It is considered a term that those drugs should be avoided or cautioned to use when the risk of adverse events may outweigh the potential benefit (Renom-Guiteras et al., 2015). Due to higher comorbidity, number of medications and lower activities of daily living (ADL), people with dementia may have higher PIM use than people without dementia (Clague et al., 2017; Raivio et al., 2006; Kristensen et al., 2018). A recent study showed that the prevalence of PIM in older people with dementia can be as high as 60% (Renom-Guiteras et al., 2018). According to systematic analyses of global disease burden in 2017, dementia is the fifth leading cause of death in China (Zhou et al., 2019). Potentially inappropriate medication (PIM) may be one of the reasons leading to death in older people with dementia, and extensive studies have also shown that the occurrence of PIM leads to an increased risk of adverse events and hospitalizations, even death (Cross et al., 2017; Wallace et al., 2017; Murphy et al., 2020). Thus, it is necessary to determine PIM use and risk factors among older patients with dementia. To date, a growing body of studies about the prevalence of PIM and risk factors have been published, but the sample size of most studies was small (Epstein et al., 2010; Fiss et al., 2013; Hanlon et al., 2015), lacking representativeness. A large sample study by Renom-Guiteras A et al. found that PIM use in older people with dementia is highly prevalent, and patients aged >8 years with more comorbidities and functional impairment are easily exposed to PIM (Renom-Guiteras et al., 2018). However, the sample of this study is limited to European countries, and the screening tool is the European Union (7)-PIM list. Due to differences in race, prescription medication and medical policy among countries, PIM use and related factors also vary based on different screening tools. In addition, there are no large sample studies on the prevalence and risk factors for PIM use in older patients with dementia in China. Therefore, we performed a large, national study to better identify PIM use and potential factors in older patients with dementia in China.

## Methods

### Setting and Sample

The cross-sectional study was carried out in eight cities of China, Chengdu, Beijing, Guangzhou, Shanghai, Shenyang, Tianjin, Zhengzhou, and Hangzhou, distributing five major geographical regions in China (east, west, north, south, central). A total of 75 hospitals were included in our study.

The participants were those diagnosed with dementia aged ≥65 years from outpatient clinics of hospitals. The diagnosis of dementia was based on the International Classification of Diseases (ICD-10) to identify Alzheimer’s disease (F000-F002, F009, G300, G301, G308 and G309), vascular dementia (F010-F013, F018 and F019), dementia in Parkinson’s disease (F023), dementia in Huntington’s disease (F022), dementia in Pick’s disease or frontotemporal dementia (F020), dementia in HIV (F024), dementia in Creutzfeldt–Jakob disease (F021) and unspecified dementias (F03X and F028). And these participants who met inclusion criteria were cluster sampled from Hospital Information System (HIS) of 75 hospitals between January 2020 and December 2020. HIS is a set of computer information management system combined with hospital management and medical activities, providing data source for numerous studies.

### Data Collection

The data from electronic medical records in outpatient clinics of 75 hospitals in eight cities were collected in three parts. The first part was patients’ sociodemographic information, including region, hospital, department, sex, and age. The second part was medical information, including disease diagnosis, payment form, and patient code. The third part was prescription information, including the generic name and trade name of medication, dosage, and the number of medications. Patients with incomplete or missing information were excluded from the study.

### Evaluation Criteria

In our study, the 2019 AGS Beers criteria were applied to detect the prevalence of PIM in older outpatients with dementia. In addition, this data information from outpatients lacks some indicators of renal function, and the rules of PIM-based eGFR (Table six in the 2019 AGS Beers criteria) were excluded. Overall, we applied Table 2 (PIM use in older adults), Table 3 (PIM use in older adults due to drug-disease or drug-syndrome interactions that may exacerbate the disease or syndrome), Table 4 (drugs used with caution in older adults), and Table 5 (potentially clinically important drug–drug interactions that should be avoided in older adults) of the 2019 AGS Beers criteria to evaluate PIM use in older people with dementia. In this study, two researchers independently analyzed and evaluated the prescription drugs per patient. The inconsistency between the two researchers was discussed by a third expert.

### Statistical Analysis

The data were analyzed by SPSS 26, and *p* value <0.05 was considered statistically significant. The results of descriptive analyses are presented as the mean ± SD (standard deviation) for continuous variables, and discontinuous variables are presented as the median ± IQR (interquartile range), frequencies or percentages. Based on PIM as a dependent variable, a binary logistic regression model was applied to identify risk factors associated with the PIM use through control covariates such as age, sex, the number of drugs, and the number of diseases.

### Ethics Approval

This study protocol was approved by the Sichuan University West China Hospital Research Ethics Board. All procedures performed in this study conformed to the standards of the 1964 Helsinki Declaration and subsequent relevant ethics.

## Results

### Basic Characteristics of Older Outpatients With Dementia

In our study, a total of 55,904 electronic medical records was extracted, 2845 incomplete or missing medical records were excluded, including 1303 items for missing gender, 1185 items for solvents, and 385 repeated drugs in a prescription. Overall, 18,624 patients with dementia were included, distributed among 75 hospitals in eight cities in China. The mean age of the study population was 80.88 ± 7.69 years, ranging from 65 to 103, and 54.85% were female. The median number of disease diagnoses was 2 (1–3), and 16.17% (3011) were diagnosed with more than five diseases (including five). Additionally, the median number of prescribed medications was 2 (1–4), and approximately 16.15% (3007) were classified as polypharmacy (defined as five or more medications). In this study, more than half of the people spent less than CNY 500 on medical care. The basic information characteristics of the population are shown in Table 1.

**TABLE 1:** Basic characteristics of older outpatients with dementia in China.

Characteristic	Chengdu			Beijing			Guangzhou			Tianjin		
	Total(N = 711)	Non-PIM (N = 445)	PIM (N = 266)	Total (N = 5086)	Non-PIM (N = 3087)	PIM (N = 1999)	Total (N = 2269)	Non-PIM (N = 1410)	PIM (N = 859)	Total (N = 1092)	Non-PIM (N = 781)	PIM (N = 311)
Sex, n (%)												
Male	344(46.98)	214(48.09)	120(45.11)	2401(47.21)	1528(49.50)	873(43.67)	1060(46.72)	703(49.86)	357(41.56)	550(50.37)	382(48.91)	168(54.02)
Female	377(53.02)	231(51.91)	146(54.89)	2685(52.79)	1559(50.50)	1126(56.33)	120(53.28)	707(50.14)	502(58.44)	542(49.63)	399(51.09)	143(45.98)
Age, n (%)												
65–80	317(44.59)	220(49.44)	97(36.47)	2361(46.42)	1521(49.27)	840(42.02)	971(42.79)	633(44.89)	338(39.35)	623(57.05)	466(59.67)	157(50.48)
>80	394(55.41)	225(50.56)	169(63.53)	2725(53.58)	1566(50.73)	1159(57.98)	1298(57.21)	777(55.11)	521(60.65)	469(42.95)	315(40.33)	154(49.52)
No. of medications n (%)												
1	143(20.11)	117(26.29)	26(9.77)	1171(23.02)	1059(34.31)	112(5.60)	421(18.55)	373(26.45)	48(5.59)	463(42.40)	447(57.23)	16(5.14)
2–4	342(48.10)	227(51.01)	115(43.23)	2822(55.49)	1585(51.34)	1237(61.88)	1279(56.37)	806(57.16)	473(55.06)	486(44.51)	273(34.96)	213(68.49)
≧5	226(31.79)	101(22.70)	125(46.99)	1093(21.49)	443(14.35)	650(32.52)	569(25.08)	231(16.38)	338(39.35)	143(13.10)	61(7.81)	82(26.37)
No. of diseases n (%)												
1–4	492(69.20)	333(74.83)	159(59.77)	3239(63.68)	2101(68.06)	1138(56.93)	2024(89.20)	1271(90.14)	753(87.66)	967(88.55)	720(92.19)	247(79.42)
5–9	173(24.33)	97(21.80)	76(28.57)	1611(31.68)	880(28.51)	731(36.57)	231(10.18)	130(9.22)	101(11.76)	116(10.62)	60(7.68)	56(18.01)
≧10	46(6.47)	15(3.37)	31(11.65)	236(4.64)	106(3.43)	130(6.50)	14(0.62)	9(0.64)	5(0.58)	9(0.82)	1(0.13)	8(2.57)
Payment n (%)												
Free	177(24.89)	111(24.94)	66(24.94)	371(7.29)	222(7.19)	149(7.45)	341(15.03)	234(16.60)	7(12.46)	1(0.09)	1(0.13)	0(0.00)
Partial Fee	452(63.57)	275(61.80)	177(53.93)	4628(90.99)	2821(91.38)	1807(90.40)	1306(57.56)	786(55.74)	520(60.54)	962(88.10)	697(89.24)	265(85.21)
Full fee	82(11.53)	59(13.26)	23(21.12)	73(1.44)	37(1.20)	36(1.80)	622(27.41)	390(27.66)	232(27.01)	106(9.71)	69(8.83)	37(11.90)
Other	0(0.00)	0(0.00)	0(0.00)	14(0.28)	7(0.23)	7(0.35)	0(0.00)	0(0.00)	0(0.00)	23(2.11)	14(1.79)	9(2.89)
No. of prescription expenditure n (%)												
<500 CNY	310(43.60)	199(44.72)	111(41.73)	1674(32.91)	1188(38.48)	486(24.31)	1455(64.13)	940(66.67)	515(59.95)	520(47.62)	388(49.68)	132(42.44)
500–1000 CNY	149(20.96)	102(22.92)	47(17.67)	1548(30.44)	941(30.48)	607(30.37)	490(21.60)	301(21.35)	189(22.00)	366(33.52)	265(33.93)	101(32.48)
>1000 CNY	252(35.44)	144(32.36)	108(40.60)	1864(36.65)	958(31.03)	906(45.32)	324(14.28)	169(11.99)	155(18.04)	206(18.86)	128(16.39)	78(25.08)

Characteristic	Shanghai			Shenyang			Hangzhou			Zhengzhou		
	Total (N = 6857)	non-PIM (N = 3786)	PIM (N = 3071)	Total (N = 433)	non-PIM (N = 293)	PIM (N = 140)	Total (N = 1052)	non-PIM (N = 696)	PIM (N = 356)	Total (N = 1124)	non-PIM (N = 782)	PIM (N = 342)
Sex, n (%)												
Male	2565(37.41)	1601(42.29)	964(31.39)	255(58.89)	17(58.70)	83(59.29)	498(47.34)	329(47.27)	169(47.47)	703(62.54)	497(63.55)	206(60.23)
Female	4292(62.59)	2185(57.71)	210(68.61)	178(41.11)	12(41.30)	57(40.71)	554(52.66)	367(52.73)	187(52.53)	421(37.46)	285(36.45)	136(39.77)
Age, n (%)												
65–80	2880(42.00)	1773(46.83)	110(36.05)	135(31.18)	10(36.18)	29(20.71)	478(45.44)	333(47.84)	145(40.73)	530(47.15)	410(52.43)	120(35.09)
≧80	3977(58.00)	2013(53.17)	196(63.95)	298(68.82)	18(63.82)	111(79.29)	574(54.56)	363(52.16)	211(59.27)	594(52.85)	372(47.57)	222(64.91)
No. of medications n (%)												
1	2982(43.49)	2403(63.47)	579(18.85)	103(23.79)	92(31.40)	11(7.86)	285(27.09)	250(35.92)	35(9.83)	164(14.59)	146(18.67)	18(5.26)
2–4	3664(53.43)	1304(34.44)	236(76.85)	164(37.88)	12(41.98)	41(29.29)	572(54.37)	358(51.44)	214(60.11)	556(49.47)	412(52.69)	144(42.11)
≧5	211(3.08)	79(2.09)	132(4.30)	166(38.34)	78(26.62)	88(62.86)	195(18.54)	88(12.64)	107(30.06)	404(35.94)	180(23.02)	224(65.50)
No. of diseases n (%)												
1–4	6744(98.35)	3730(98.52)	3014(98.14)	310(71.59)	232(79.18)	78(55.71)	897(85.27)	594(85.34)	303(85.11)	940(83.63)	690(88.24)	250(73.10)
5–9	111(1.62)	55(1.45)	56(1.82)	106(24.48)	53(18.09)	53(37.86)	143(13.59)	96(13.79)	47(13.20)	162(14.41)	80(10.23)	82(23.98)
≧10	2(0.03)	1(0.03)	1(0.03)	17(3.93)	8(2.73)	9(6.43)	12(1.14)	6(0.86)	6(1.69)	22(1.96)	12(1.53)	10(2.92)
Payment n (%)												
Free	3329(48.55)	1277(33.73)	205(66.82)	296(68.36)	19(64.85)	106(75.71)	38(3.61)	21(3.02)	17(4.78)	161(14.32)	81(10.36)	80(23.39)
Partial Fee	3147(45.89)	2316(61.17)	831(27.06)	114(26.33)	85(29.01)	29(20.71)	970(92.21)	642(92.24)	328(92.13)	639(56.85)	475(60.74)	164(47.95)
Full fee	375(5.47)	191(5.04)	184(5.99)	23(5.31)	18(6.14)	5(3.57)	38(3.61)	29(4.17)	9(2.53)	324(28.83)	226(28.90)	98(28.65)
Other	6(0.09)	2(0.05)	4(0.13)	0(0.00)	0(0.00)	0(0.00)	6(0.57)	4(0.57)	2(0.56)	0(0.00)	0(0.00)	0(0.00)
No. of prescription expenditure n (%)												
<500 CNY	5288(77.12)	2842(75.07)	244(79.65)	117(27.02)	97(33.11)	20(14.29)	483(45.91)	336(48.28)	147(41.29)	491(43.68)	352(45.01)	139(40.64)
500–1000 CNY	1182(17.24)	704(18.59)	478(15.56)	125(28.87)	97(33.11)	28(20.00)	294(27.95)	194(27.87)	100(28.09)	419(37.28)	284(36.32)	135(39.47)
>1000 CNY	387(5.64)	240(6.34)	147(4.79)	191(44.11)	99(33.79)	92(65.71)	275(26.14)	166(23.85)	109(30.62)	214(19.04)	146(18.67)	68(19.88)

### The Prevalence of PIMs and Leading Medications

**FIGURE 1:**
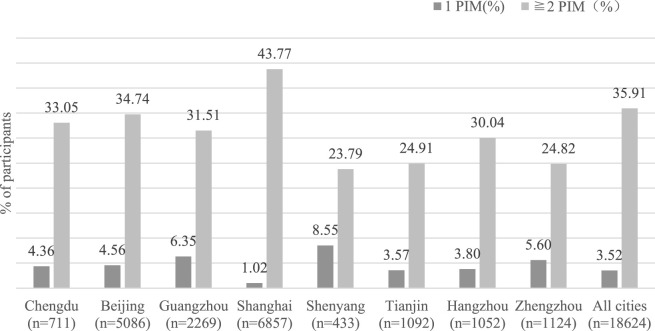
Prescription of 1 PIM and ≥2 PIMs among older outpatients with dementia in China.

Of 18,624 older patients with dementia, 3.52% (656) were detected with 1 PIM, and 35.91% (6688) received at least two PIMs. The prevalence in the eight cities ranged from 28.48 to 44.79%. The prevalence of PIMs in eight cities is displayed in Figure 1.

**FIGURE 2:**
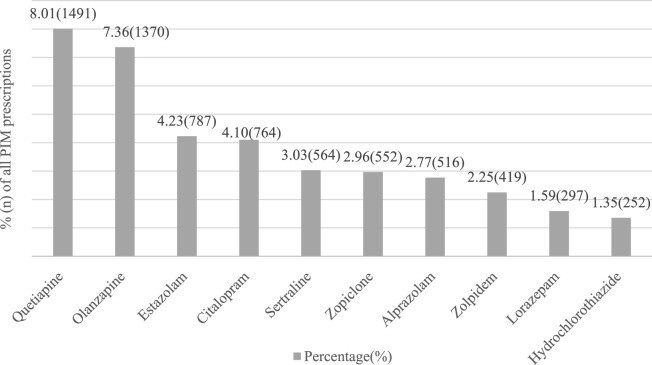
Ten most frequently prescribed PIMs among older outpatients with dementia in China.

According to the 2019 AGS Beers criteria, the antipsychotic drugs quetiapine and olanzapine were most frequently prescribed in older outpatients with PIM, accounting for 8.01% (1491) and 7.36% (1370), respectively. In addition, SSIRs (citalopram, sertraline) and sedative hypnotics (estazolam, zopiclone, alprazolam, zolpidem, lorazepam) were also observed in top drugs of PIM. Figure 2 lists the percentages and names of the top ten drugs.

### Risk Factors Associated With PIM

Table 2 displays the results of the multiple logistic regression. Considering PIM to be a dependent variable, age >80 years, the number of medications was associated with the occurrence of PIM. In addition, females with dementia were likely to receive PIM. In our study, we also found that the payment form negatively affected PIM.

**TABLE 2:** Multivariable analysis of risk factors associated with PIM among older outpatients with dementia in China.

Variables	Odds Ratio	CI Lower	CI Upper	*p* Value
Sex, n (%)				
Female	Reference			
Male	0.892	0.839	0.947	<0.001
Age, n (%)				
65–80	Reference			
≧80	1.074	1.011	1.142	0.021
No. of medications n (%)				
1	Reference			
2–4	1.22	1.137	1.308	<0.001
≧5	1.118	1.001	1.249	0.048
No. of diseases n (%)				
1–4	Reference			
5–9	0.951	0.866	1.045	0.296
≧10	0.73	0.577	0.923	0.009
Payment n (%)				
Full fee	Reference			
Partial Fee	0.463	0.432	0.497	<0.001
Free	0.488	0.433	0.549	<0.001
others	0.284	0.147	0.546	<0.001
No. of prescription expenditure n (%)				
<500 CNY	Reference			
500–1000 CNY	0.893	0.827	0.964	0.004
>1000 CNY	1.02	0.934	1.113	0.666

## Discussion

In our study, a total of 18,624 participants in eight cities in China were recruited, which is larger than a previous study in China, making our results more representative. The cross-sectional study revealed that PIM in older outpatients with dementia is highly prevalent and identified three potential factors increasing the number of PIMs. Links exist between PIM and drug-related problems (Hagstrom et al., 2015; Muhlack et al., 2017), and the connection is more apparent in older people. Based on these results, resolving related medical problems may be useful.

The prevalence of PIM in our study was 39.43%, and 35.91% were prescribed at least two PIMs. Compared with the Ferreira et al. study using the 2019 AGS Beers criteria, our result was lower (Ferreira et al., 2021). The study population and sample size may be responsible for the difference. While the prevalence of PIM reported by Cross et al. was lower than our study results (Cross et al., 2016), it was possibly due to the difference in PIM screening tools. In our study, we applied the 2019 AGS Beers criteria to determine PIM, while Cross et al.‘s study only used dementia-specific PIMs of the Beers criteria. In addition, the prevalence of PIM varied in the regions included in the study, ranging from 28.48 to 44.79%. Figure 1 shows that Tianjin was the city with the lowest percentage of PIM. The lower number of drug medications per dementia patient in Tianjin may be the explanation of the phenomenon. In addition, the prevalence of PIM in Shanghai ranked first in eight cites, and several reasons may be responsible. First, the difference among PIM was attributed to the sample size. Of the total population included, more than one-third of participants were from Shanghai. Second, the difference in medication habits among clinicians may explain the gap in PIM use. According to the dataset comprising dementia patients from eight cities, the frequency of quetiapine use (ranking first in prescribed PIM prescriptions) in Shanghai accounted for 68.61% (1023/1491) of the total amount of quetiapine.

In our study, we found that antipsychotics were frequently prescribed by clinicians in dementia patients with PIM, similar to Renom-Guitera et al.‘s study. In addition, the number of quetiapine was higher than olanzapine, ranking first in the top ten PIMs according to the 2019 AGS Beers criteria, consistent with the study by Machado-Duque et al. (Machado-Duque et al., 2021). In contrast, olanzapine was prescribed most frequently in the United States (Maust et al., 2015). It is possible that the larger number of dementia patients with Parkinson’s disease in China leads to more prescriptions of quetiapine because olanzapine should be avoided in PD patients due to the adverse effects of the extrapyramidal system (Zhang et al., 2005; Li et al., 2019; Aarsland et al., 2005; American Geriatrics Society Beers Criteria^®^ Update Expert Panel, 2019). It is worth noting that the number of antipsychotic drugs prescribed was much higher than that of other medications among the top ten PIMs. This frequent use of antipsychotic drugs may be compatible with dementia patients who always had accompanying mental symptoms such as agitation and aggressiveness. A study by Calsolaro et al. also pointed out that it is sometimes necessary to use antipsychotics to control symptoms (Calsolaro et al., 2021). Due to the side effects of antipsychotic drugs, such as the decline in cognitive function, cerebrovascular events, severe extrapyramidal effects and mortality (American Geriatrics Society Beers Criteria^®^ Update Expert Panel, 2019; Calsolaro et al., 2021; Vinaşi et al., 2021), clinicians should be cautious when prescribing treatment for patients with dementia.

Benzodiazepines (sedative hypnotics) are frequently prescribed by clinicians to treat patients with sleep disorders. Sleep disorders can be observed in older adults, especially people with dementia (Tian et al., 2017; Gulia et al., 2018; Ward et al., 2020). In our study, estazolam, a benzodiazepine drug, was the third most commonly used PIM after quetiapine and olanzapine, accounting for 4.23% (787). The higher prescription rate could be attributed to the higher prevalence of sleep disorders in older patients with dementia. However, prolonged use of benzodiazepine may result in a series of adverse effects, such as falls, cognitive decline, and mortality (Barker et al., 2004; Pek et al., 2017). International guidelines suggest that older people with dementia should avoid the use of benzodiazepines as much as possible. Thus, benzodiazepines are taken into consideration when clinicians comprehensively evaluate the condition of patients and there is no alternative drug treatment.

According to a binary logistic model, our study identified three risk factors associated with PIM: female sex, number of medications, and increasing age. Aging is inextricably linked to the deterioration of organ function, causing alterations in pharmacokinetics and pharmacodynamics and further causing some drug-related problems (Fried et al., 2014; Payne et al., 2016). To our knowledge, 80 years was considered a cutoff of advanced age. Compared with those aged 65–79 years, those aged >80 years easily suffered more prescription and PIM (Mo et al., 2016). In our study, age> 80 years was also considered a potential risk factor, consistent with an earlier published study (Murphy et al., 2020). Increased PIMs in females may be due to the following reasons: 1) the risk diagnosed with dementia in women was higher than men; 2) women focus more on their health issue and have more healthcare visits and complaints; 3) they are more likely to use psychotropic drugs with anticholinergic properties compared to men (Bierman et al., 2007; Johnell et al., 2009; Jia et al., 2020). Regarding the number of medications, the more drugs you take, the more likely you are to have PIM. The strong association between the number of medications and PIM has been confirmed by numerous studies, consistent with our study results (Ma et al., 2018; Tian et al., 2021). Interestingly, we found that reimbursement was negatively related to the occurrence of PIM. This may be due to reimbursement making it less expensive to have more drug options.

There are strengths and limitations in our study. First, a large samplein eight cities in China is our strengths, making the results more reliable. Second, it made up for the lack of PIM and risk factors in patients with dementia in China. However, this was a cross-sectional study, which is prone to bias the results. And some unmeasured confounding factors and lacking follow-up and other medical data might make the related risk factors not be analyzed comprehensively. In our study, we just applied Table 2, 3, 4,5 of 2019 AGS Beers criteria, Table 6 was excluded due to the absence of renal indicators. The prevalence of PIM in our study may be underestimated. Additionally, participants with dementia in this study were outpatients, which was not sicker than inpatients, the finding might not apply to inpatients. Therefore, we need to further carry out a study among inpatients or nursing home patients with dementia and collected related follow-up data.

## Conclusion

The current study shows that the prevalence of PIM among outpatients with dementia in China is high. In addition, age >80 years, female sex, and taking multiple medications are risk factors for an increasing number of PIMs. Notably, among patients with PIM, antipsychotic drugs were the most frequent and much more frequent than other drugs. This prompted us to explore the use of antipsychotics in dementia patients and the relationship between antipsychotics and adverse reactions in patients with dementia in further research.

## Data Availability

The original contributions presented in the study are included in the article/Supplementary Material, further inquiries can be directed to the corresponding author.
